# Cardiovascular Outcomes in the Patients With Colorectal Cancer: A Multi-Registry-Based Cohort Study of 197,699 Cases in the Real World

**DOI:** 10.3389/fcvm.2022.851833

**Published:** 2022-06-16

**Authors:** Shilong Zhang, Yan Wang, Pengfei Zhang, Luoyan Ai, Tianshu Liu

**Affiliations:** ^1^Department of Medical Oncology, Zhongshan Hospital, Fudan University, Shanghai, China; ^2^Centre for Evidence-Based Medicine, Fudan University, Shanghai, China

**Keywords:** SEER database, cardiovascular death, competing-risk model, nomogram, cause-specific death, colorectal cancer

## Abstract

**Purpose:**

We aimed to investigate the mortality patterns and quantitatively assess the risks of cardiovascular death (CVD) in patients with colorectal cancer (CRC). We also established a competing-risk model to predict the probability of CVD for patients with CRC.

**Patients and Methods:**

Patients with CRC who diagnosed between 2007 and 2015 in the Surveillance, Epidemiology, and End Results (SEER) database were included in the present study. The cumulative incidence function (CIF) was used for CVD and other causes of death, and Gray’s test was used to determine the subgroup difference in CIF. The Fine-Gray proportional subdistribution hazards model was used for identifying independent risk factors for CVD. A novel competing-risk model was established to evaluate the probability of CVD for patients with CRC. The performance of the nomogram was measured by concordance index (C-index), calibration curve, decision curve analysis (DCA), and risk stratification.

**Results:**

After a median follow-up of 37.00 months, 79,455 deaths occurred, of whom 56,185 (70.71%) succumbed to CRC and 23,270 (29.29%) patients died due to non-CRC, among which CVD accounted for 9,702 (41.69%), being the major cause of non-cancer deaths. The 1-, 3-, and 5-year cumulative rates for CVD were 12.20, 24.25, and 30.51%, respectively. In multivariate analysis, age, race, marital status, tumor size, tumor stage, advanced stage, surgery, and chemotherapy were independent risk factors of CVD among patients with CRC. The nomogram was well calibrated and had good discriminative ability, with a c-index of 0.719 (95% CI, 0.738–0.742) in the training cohort and 0.719 (95% CI, 0.622–0.668) in the validation cohort. DCA demonstrated that nomogram produced more benefit within wide ranges of threshold probabilities for 1-, 3-, and 5-year CVD, respectively.

**Conclusion:**

This study was the first to analyze the CIF and risk factors for CVD among CRC based on a competing-risk model. We have also built the first 1-, 3-, and 5-year competing nomogram for predicting CVD. This nomogram had excellent performance and could help clinicians to provide individualized management in clinical practice.

## Introduction

Colorectal cancer (CRC), a common gastrointestinal cancer, is ranked as one of the three most common cancers worldwide, with 1,147,950 new cases and 53,200 deaths estimated in 2020 (1). The life expectancy of patients with CRC has considerably improved due to early diagnosis and treatment (2, 3). Therebefore, increasing mortality burden is not derived from cancer but from non-cancer causes. However, the political risk of non-cancer mortality is an objective existence, but it hasn’t caused plenty of attention in academia.

In the past decade, cardiovascular death (CVD) has been regarded as one of the most common late complications of cancer therapy (4, 5). Indeed, the introduction of novel chemotherapeutic or immunotherapeutic drugs has brought considerable survival benefits for patients with advanced tumors (6). Unfortunately, these agents can cause a series of adverse events in clinical practice (7–10), mostly due to the induced overactivation of immunity or even direct killing of non-target organs, including the heart (11, 12). Therefore, it is an emerging issue that warrants increased awareness and investigation by cardiologists, oncologists, and immunologists.

Despite multiple studies showing how chemotherapeutic and immunotherapeutic drugs may contribute to the increased risk of CVD among cancer survivors, studies that focus on the cardiovascular outcomes in patients with CRC remain scarce. A prior descriptive analysis of Surveillance, Epidemiology, and End Results (SEER) data reported that patients with CRC were associated with an increased risk of CVD within 1 year of diagnosis. Although this analysis highlights the frequency of CVD among CRC survivors (13), it has mainly focused on CVD using the standard Cox proportional hazards regression approach. This conventional method might lead to unreliable results when competing events are present (14). Competing risks usually exist in the field of medicine, which may sway the occurrence of endpoint events. In addition, they would become particularly critical in terms of the elderly population or long prognosis (15). Thus, competing risks is certainly worth taking into consideration when investigating the CVD of patients with CRC, which would give a clearer picture of CVD risks that these patients would confront.

In the present study, we aimed to perform a population-based analysis of a cohort of patients with CRC between 2007 and 2015 in the SEER database to identify the risk factors for CVD among patients with CRC, including those within different subgroups. Since competitive events exist when analyzing CVD through Cox regression model (16), we used a competitive risk model when working for this type of data and objective of study. We comprehensively assessed the risks of CVD among more than 42,000 patients with CRC. Based on these results, we built and internally validated a competing-risk model to evaluate the probabilities of CVD for patients with CRC. Our findings can help clinicians adopt accurate risk stratification, weigh the advantages and disadvantages of therapies, and help with the cure of disease, improve the prognosis, and raise the quality of life for patients with CRC.

## Materials and Methods

### Data Source and Study Cohort

The present study was a retrospective analysis of a cohort of patients with CRC that strictly followed the Strengthening the Reporting of Observational Studies in Epidemiology (STROBE) specifications (17). The data used in this study were taken from 18 SEER registries *via* the SEER*Stat software (2017 submission). The 18 SEER registries with additional treatment fields that were used in this study provided detailed data about demographic and clinicopathological characteristics, treatment protocols, and follow-up.

### Study Population and Variables

This study included cases from the 18 SEER registries with CRC, which were proved by pathologic diagnosis. We selected the patients with CRC using the following topography codes: C18.0, C18.2, C18.3, C18.4, C18.5, C18.6, C18.7, C18.8, 18.9, C19.9, and C20.9. The dawn of tyrosine kinase inhibitors, 2007, was selected as a year of insurance that was accessible in the SEER database when investigating the role of socioeconomic factors in CVD (18). The eligible patients were selected using the following inclusion criteria: (1) diagnosed with CRC as the primary and only tumor; (2) diagnosed between 2007 and 2015; and (3) had active follow-up information and defined causes of mortalities. Then, the following information was obtained for each patient: year of diagnosis, age, sex, race, tumor stage, histological grade, tumor site, marital status, insurance status, surgery, radiotherapy, chemotherapy, survival months, and causes of death. Patients with missing data about any of above information were excluded.

### Outcomes

Cardiovascular death was the primary endpoint and was measured as the time from the date of diagnosis of CRC to death due to CVD (19). As recorded in the SEER database, CVD has six causes of death, namely, disease of heart, hypertension without heart disease, cerebrovascular disease, atherosclerosis, aortic aneurysm and dissection, and other diseases of arteries, arterioles, and capillaries (20). The non-CVD was defined from other causes and was considered as competing events against CVD. Patients who survived until the last follow-up or who were lost to follow-up before the end of the observation period were regarded as censored observations.

### Statistical Analysis

The difference of baseline characteristics between subgroups were compared with χ^2^. Cumulative incidence function (CIF) was calculated to evaluate the probabilities of each event at 1-, 3-, and 5-year. Subgroup analyses were performed based on patient’s characteristics, and respective curves for CIF were produced. The difference in CIF were determined through Gray’s test (21). Fine and Gray’s subdistribution proportional hazards model was performed for identifying the independent risk factors for CVD among patients with CRC (22). Moreover, based on Fine-Gray’s model, a novel competing-risk model was developed to predict the probabilities of CVD at 1-, 3-, 5-year for patients with CRC. We used the concordance index (C-index) to measure discriminative performance of the model, and the consistency was measured using a calibration curve (18). Decision curve analysis (DCA) was performed to visually investigate the clinical utilities and net benefits of this model (23, 24). The packages *cmprsk*, *survival*, *mstate*, *rm*s, *pec*, and *riskRegression* in the R software (version 3.2.5) were used to establish and validate the nomogram. *p* < 0.05 in two-sided tests were statistically significant.

## Results

### Patient Selections and Baseline Characteristics

Our study extracted 197,699 eligible patients diagnosed with CRC between 2007 and 2015 in the SEER program. The baseline characteristics of the whole study cohort are presented in Table 1. A larger proportion of patients were aged above 65 years (1,776, 65.7%), male (102,669, 51.9%), white (156,015, 78.9%), married (111,210, 56.3%), and insured (165,937, 83.9%). Most patients were diagnosed with grade II (69.6%), followed by grade III (16.8%), grade I (10.9%), and grade IV (2.8%) CRC. The distribution of SEER stage was as follows: 77,655 (39.3%) had localized stage, 81,662 (41.3%) had regional stage, and 38,382 (19.4%) had distant stage. A total of 179,100 (90.6%) patients were treated with surgery, 81,946 (41.4%) patients were treated with chemotherapy, and only 28,737 (14.5%) patients were treated with radiotherapy.

**TABLE 1:** Demographic and clinicopathological characteristics of the included CRC patients.

Characteristics	Number (%)
**Total**	197,699
**Year of diagnosis**	
2007–2010	89,006 (45.0)
2011–2015	108,693 (55.0)
**Age**	
<65	98,682 (49.9)
≥65	99,017 (50.1)
**Sex**	
Female	95,030 (48.1)
Male	102,669 (51.9)
**Race**	
Black	23,557 (11.9)
White	156,015 (78.9)
Other	18,127 (9.2)
**Marital status**	
Married	111,210 (56.3)
Unmarried	86,489 (43.7)
**Insurance**	
Any Medicaid	24,892 (12.6)
Insured	165,937 (83.9)
Uninsured	6,870 (3.5)
Tumor site	
Left	101,144 (51.2)
Right	93,632 (47.4)
NOS	2,923 (1.5)
Tumor size	
≤5 cm	112,065 (56.7)
5–10 cm	52,078 (26.3)
>10 cm	33,556 (17.0)
**Grade**	
Grade I	21,461 (10.9)
Grade II	137,501 (69.6)
Grade III	33,300 (16.8)
Grade IV	5,437 (2.8)
**SEER stage**	
Localized	77,655 (39.3)
Regional	81,662 (41.3)
Distant	38,382 (19.4)
**Surgery**	
No	18,599 (9.4)
Yes	179,100 (90.6)
**Radiotherapy**	
No	168,962 (85.5)
Yes	28,737 (14.5)
**Chemotherapy**	
No	115,753 (58.6)
Yes	81,946 (41.4)
**Causes**	
Alive	118,244 (59.8)
Death form CRC	9,702 (4.9)
Death form CVD	56,185 (28.4)
Death form non-CVD	13,568 (6.9)

*Other, American Indian/Alaska Native/Asian/Pacific Islander; NOS, not otherwise specified; SEER, Surveillance, Epidemiology, and End Results; CRC, colorectal cancer; CVD, cardiovascular death.*

### Cumulative Incidence Function Survival Analysis

**FIGURE 1:**
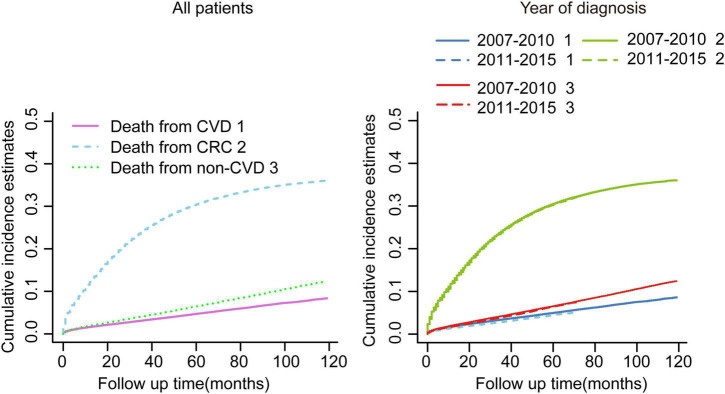
Cumulative incidence estimates of cardiovascular death (CVD) among patients with colorectal cancer (CRC) in the whole cohort. Death from CVD was indicated as 1; death from CRC was indicated as 2; and death from non-CVD was indicated as 3.

The median follow-up of the whole cohort was 37 months (IQR: 17.00–119.00). In total, 79,455 patients died during the follow-up, of whom 56,185 (70.71%) succumbed to CRC and 23,270 (29.29%) died due to non-CRC, among which CVD accounted for 9,702 (41.69%), being the major cause of non-cancer deaths (Table 1). In consideration of competing risks (death from other causes), we further performed cumulative incidence analysis in the whole cohort (Table 2). Overall, the 1-, 3-, and 5-year CIF of death due to CRC were 1.63, 3.16, and 4.71%, respectively. The 1-, 3-, and 5-year CIF of CVD were 12.20, 24.25, and 30.51%, respectively, while the 1-, 3-, and 5-year CIF of non-CVD were 1.93, 4.13, and 4.77%, respectively (Table 2). Furthermore, the CIF of CVD were significantly decreased in recent years (Figure 1 and Table 2).

**TABLE 2:** Cumulative incidence of cause-specific death and Gray’s test in the whole set.

Characteristics	CVD (%)			*P*	Non-CVD (%)			*P*
	1-year	3-year	5-year		1-year	3-year	5-year	
Total	12.20	24.25	30.51		1.93	4.13	4.77	
Year of diagnosis				<0.001				0.01
2007–2010	1.84	3.47	4.99		2.10	4.30	6.60	
2011–2015	1.46	2.89	4.49		1.79	3.99	6.45	
Age				<0.001				<0.001
<65	0.44	0.94	1.39		0.87	1.95	2.99	
≥65	2.82	5.34	7.89		2.97	6.26	9.79	
Sex				0.210				0.010
Female	1.55	3.03	4.65		1.83	3.95	6.27	
Male	1.71	3.28	4.77		2.01	4.31	6.67	
Race				<0.001				<0.001
Black	1.72	3.23	4.46		1.99	4.27	6.35	
White	1.68	3.26	4.91		2.00	4.26	6.70	
Others	1.14	2.19	3.20		1.20	2.87	4.65	
Marital status				<0.001				<0.001
Married	1.23	2.40	3.63		1.46	3.26	5.31	
Unmarried	2.15	4.15	6.13		2.53	5.26	8.00	
Insurance status				<0.001				<0.001
Any Medicaid	1.89	3.65	5.16		2.63	5.33	7.66	
Insured	0.70	1.43	2.14		1.29	2.78	3.62	
Uninsured	1.63	3.16	4.75		1.85	4.01	6.42	
Tumor site				<0.001				<0.001
Left	1.34	2.65	3.89		1.55	3.40	5.32	
Right	1.94	3.69	5.59		2.30	4.90	7.71	
NOS	2.07	3.82	4.97		3.20	5.05	6.79	
Tumor size				<0.001				<0.001
≤5 cm	1.53	3.14	4.84		1.77	4.16	6.73	
5–10 cm	1.79	3.30	4.78		2.07	4.19	6.40	
>10 cm	1.72	3.02	4.18		2.22	3.97	5.76	
Grade				<0.001				<0.001
Grade I	1.22	2.86	4.58		1.64	3.90	6.46	
Grade II	1.64	3.20	4.83		1.86	4.17	6.59	
Grade III	1.86	3.18	4.34		2.25	4.08	6.06	
Grade IV	1.79	3.29	4.50		2.61	4.51	6.19	
SEER stage				<0.001				<0.001
Localized	1.71	3.72	5.93		1.99	4.81	8.08	
Regional	1.72	3.22	4.75		1.98	4.20	6.52	
Distant	1.29	1.91	2.17		1.69	2.63	3.15	
Surgery				<0.001				<0.001
No	2.48	3.55	4.13		3.05	4.55	5.40	
Yes	1.55	3.12	4.77		1.81	4.09	6.59	
Radiotherapy				<0.001				<0.001
No	1.78	3.42	5.08		2.09	4.42	6.90	
Yes	0.77	1.66	2.53		0.97	2.47	4.00	
Chemotherapy				<0.001				<0.001
No	2.36	4.47	6.62		2.74	5.60	8.74	
Yes	0.61	1.33	2.02		0.78	2.07	3.29	

*Other, American Indian/Alaska Native/Asian/Pacific Islander; NOS, not otherwise specified; SEER, Surveillance, Epidemiology, and End Results.*

**FIGURE 2:**
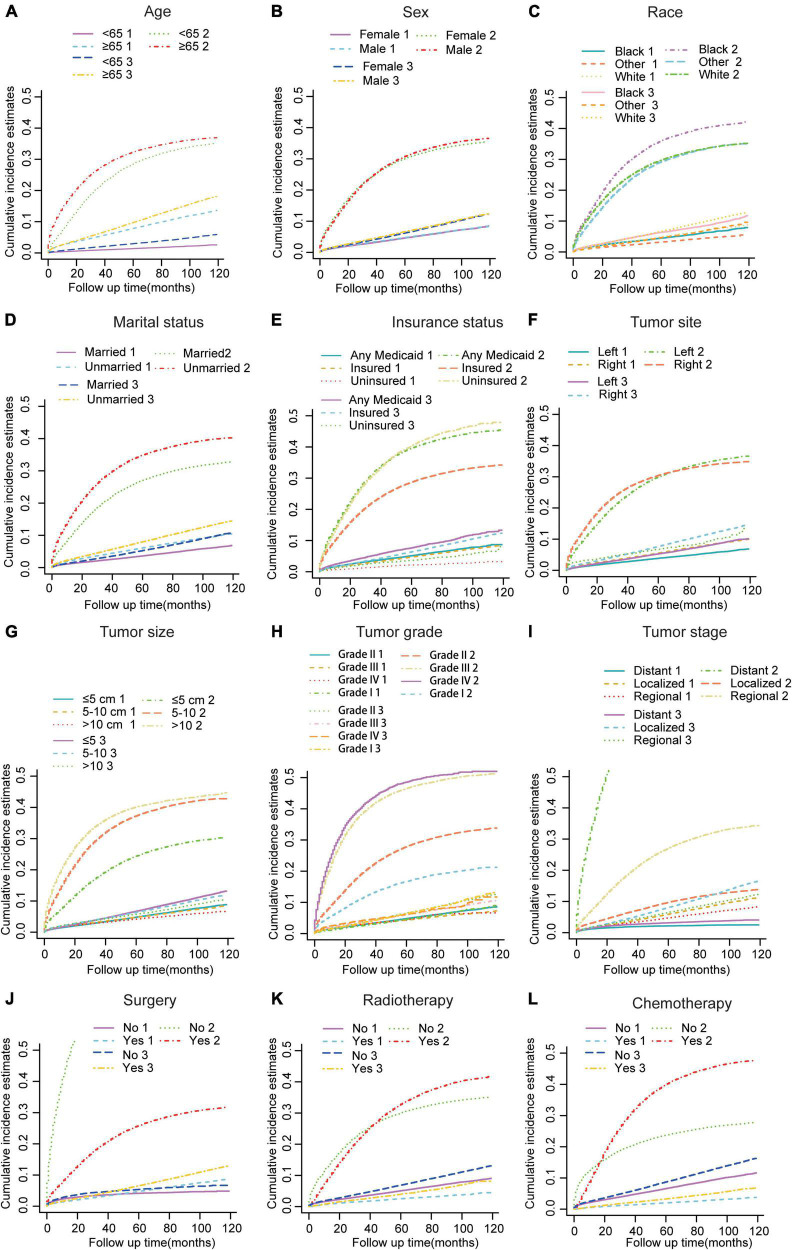
Cumulative incidence estimates of CVD among patients with CRC according to **(A)** Age; **(B)** Sex; **(C)** Race; **(D)** Marital status; **(E)** Insurance status; **(F)** Tumor site; **(G)** Tumor size; **(H)** Grade; **(I)** SEER stage; **(J)** Surgery; **(K)** Radiotherapy; **(L)** and Chemotherapy. A solid line represents cause-specific death, while a dotted line represents other causes of death. Death from CVD was indicated as 1; death from CRC was indicated as 2; and death from non-CVD was indicated as 3.

In the subsequent subgroup analyses stratified by patient characteristics (Table 2), we found that a high CVD primarily occurred in patients aged ≥65 years (Figure 2A) whose race was White (Figure 2C); were unmarried (Figure 2D); had any Medicaid (Figure 2E); who had right tumors (Figure 2F), small tumor size (Figure 2G), and I-II grade of tumor (Figure 2H); had localized SEER stage (Figure 2I); and were not treated with surgery (Figure 2J), radiotherapy (Figure 2K), or chemotherapy (Figure 2L). In addition, no significant difference in CVD was found in sex subgroup analyses (Figure 2B).

### Risk Factors for Cardiovascular Death Among Patients With Colorectal Cancer in the Training Cohort

As shown in Table 3, patients with CRC in the whole cohort were randomized into the training (*n* = 138,391) and validation cohort (*n* = 59,308) at a ratio of 7:3. The baseline characteristics between the two cohorts were well balanced. Furthermore, the CIF of CVD remained comparable between the two cohorts (*p* = 0.57). To identify the independent risk factors for CVD in the training cohort, we conducted the univariate and multivariate Fine-Gray hazard model analysis. The univariate analysis showed that CVD was significantly associated with age, race, marital status, insurance status, tumor site, tumor size, SEER stage, surgery, radiotherapy, and chemotherapy (Table 4). However, sex and grade did not significantly influence cumulative incidences of CVD. To minimize the risks of producing false positive results, multivariate analyses based on Fine-Gray hazard model were conducted to control the significant covariates. Results showed that age, race, marital status, tumor size, SEER stage, surgery, and chemotherapy were independent risk factors for CVD (Table 4).

**TABLE 3:** Basic characteristics of patients in the training and validation cohorts.

Characteristics	Training cohort *N* (%)	Validation cohort *N* (%)	*P*
**Total**	**138,391 (100)**	**59,308 (100)**	

Age			0.217
<65	68,952 (49.8)	29,730 (40.1)	
≥65	69,439 (50.2)	29,578 (49.9)	
Sex			0.442
Female	66,443 (48.0)	28,587 (48.2)	
Male	71,948 (52.0)	30,721 (51.8)	
Race			0.488
Black	16,569 (12.0)	6,988 (11.8)	
White	109,146 (78.9)	46,869 (79.0)	
Other	12,676 (9.2)	5,451 (9.2)	
Year of diagnosis			0.499
2007–2010	62,374 (45.1)	26,632 (44.9)	
2011–2015	76,017 (54.9)	32,676 (55.1)	
Marital status			0.305
Married	78,031 (56.4)	33,179 (55.9)	
Unmarried	60,360 (43.6)	26,129 (44.1)	
Insurance			0.962
Any Medicaid	17,421 (12.6)	7,471 (12.6)	
Insured	116,171 (83.9)	49,766 (83.9)	
Uninsured	4,799 (3.5)	2,071 (3.5)	
Tumor site			0.248
Left	70,638 (51.0)	30,506 (51.4)	
Right	65,713 (47.5)	27,919 (47.1)	
NOS	2,040 (1.5)	883 (1.5)	
Tumor size			0.761
≤5 cm	78,372 (56.6)	33,693 (56.8)	
5–10 cm	36,503 (26.4)	15,575 (26.3)	
>10 cm	23,516 (17.0)	10,040 (16.9)	
Grade			0.238
Grade I	15,014 (10.8)	6,447 (10.9)	
Grade II	96,153 (69.5)	41,348 (69.7)	
Grade III	23,354 (16.9)	9,946 (16.8)	
Grade IV	3,870 (2.8)	1,567 (2.6)	
SEER stage			0.204
Localized	54,182 (39.2)	23,473 (39.6)	
Regional	57,291 (41.4)	24,371 (41.1)	
Distant	26,918 (19.5)	11,464 (19.3)	
Surgery			0.711
No	13,042 (9.4)	5,557 (9.4)	
Yes	125,349 (90.6)	53,751 (90.6)	
Radiotherapy			0.273
No	118,354 (85.5)	50,608 (85.3)	
Yes	20,037 (14.5)	8,700 (14.7)	
Chemotherapy			0.434
No	80,949 (58.5)	34,804 (58.7)	
Yes	57,442 (41.5)	24,504 (41.3)	
Death causes			0.267
Alive	82,788 (59.8)	35,456 (59.8)	
Death form CRC	39,417 (28.5)	16,768 (28.3)	
Death form CVD	6,712 (4.9)	2,990 (5.0)	
Death from non-CVD	9,474 (6.8)	4,094 (6.9)	

*Other, American Indian/Alaska Native/Asian/Pacific Islander; NOS, not otherwise specified; SEER, Surveillance, Epidemiology, and End Results; CRC, colorectal cancer; CVD, cardiovascular death.*

**TABLE 4:** Univariate and multivariable competing risk analyses for cardiovascular death (CVD) among patients with colorectal cancer (CRC) in the training cohort.

	Univariate analysis		Multivariate analysis	
Variables	sdHR (95% CI)	*P*	sdHR (95% CI)	*P*
**Age**				
<65	Reference		Reference	
≥65	5.80 (5.42–6.21)	<0.001	4.65 (4.34–4.99)	<0.001
**Sex**				
Female	Reference			
Male	1.04 (0.99–1.10)	0.077		
**Race**				
Black	Reference		Reference	
White	1.07 (0.993–1.15)	0.07	0.94 (0.87–1.01)	0.095
Others	0.70 (0.62–0.78)	<0.001	0.67 (0.60–0.76)	<0.001
**Marital status**				
Married	Reference		Reference	
Unmarried	1.63 (1.55–1.71)	<0.001	1.33 (1.26–1.40)	<0.001
**Insurance**				
Insured	Reference		Reference	
Any Medicaid	2.81 (2.26–3.51)	<0.001	1.25 (1.00–1.56)	0.051
Uninsured	2.65 (2.14–3.27)	<0.001	1.01 (0.81–1.25)	0.940
**Tumor site**				
Left	Reference		Reference	
Right	1.10 (0.90–1.33)	0.35	0.96 (0.79–1.17)	0.710
NOS	0.75 (0.62–0.91)	0.003	0.94 (0.77–1.14)	0.520
Tumor size				
≤5 cm	Reference		Reference	
5–10 cm	1.01 (0.96–1.07)	0.710	1.07 (1.01–1.14)	0.017
>10 cm	0.85 (0.80–0.91)	<0.001	0.99 (0.92–1.07)	0.810
**Grade**				
Grade I	Reference			
Grade II	1.016 (0.94–1.10)	0.69		
Grade III	0.94 (0.86–1.04)	0.22		
Grade IV	0.86 (0.72–1.02)	0.08		
**SEER stage**				
Localized	Reference		Reference	
Regional	0.786 (0.75–0.83)	<0.001	0.99 (0.94–1.05)	0.930
Distant	0.334 (0.31–0.37)	<0.001	0.47 (0.43–0.52)	<0.001
**Surgery**				
No	Reference		Reference	
Yes	1.24 (1.13–1.36)	<0.001	0.81 (0.72–0.90)	<0.001
**Radiotherapy**				
No	Reference		Reference	
Yes	0.47 (0.43–0.52)	<0.001	1.01 (0.91–1.13)	0.780
**Chemotherapy**				
No	Reference		Reference	
Yes	0.30 (0.28–0.32)	<0.001	0.48 (0.45–0.52)	<0.001

*sdHR, subdistribution hazard ratio; CI, confidence interval; Other, American Indian/Alaska Native/Asian/Pacific Islander; NOS, not otherwise specified; SEER, Surveillance, Epidemiology, and End Results; CRC, colorectal cancer; CVD, cardiovascular death.*

### Construction of a Competing-Risk Model

**FIGURE 3:**
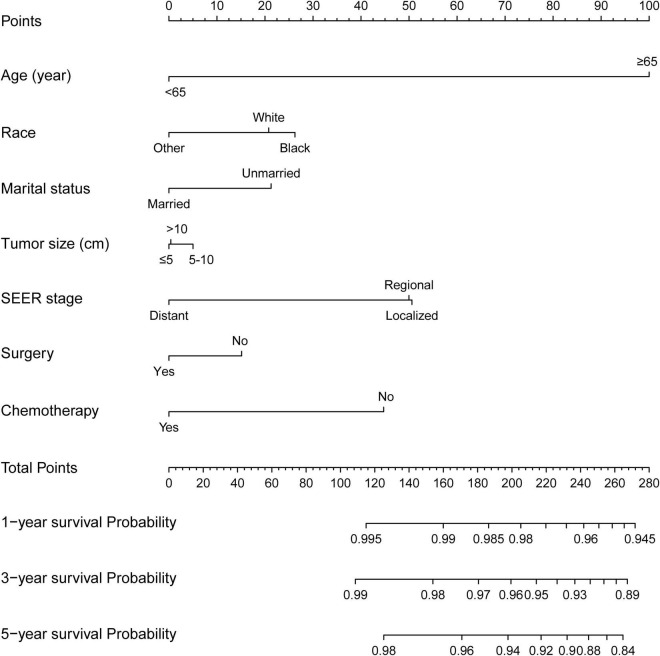
Competing-risk model for predicting the 1-, 3-, and 5-year probabilities of CVD among patients with CRC. The “total points” of a certain patient was calculated by adding all the scores of the 7 parameters. Based on the total points, the possibilities of CVD at different timepoints and the prognostic group was obtained.

The incidence of CVD in patients with CRC has tended to increase in the last decades. However, competing-risk model combining comprehensive factors for patients with CRC suffering CVD remains scarce. Thus, a nomogram predicting the probabilities of CVD at 1-, 3-, and 5-year was established (Figure 3) based on the Fine and Gray’s model we built. With the help of this useful tool, an individual patient chance of CVD at different times could be easily obtained by adding the scores of each incorporated variable.

### Validation and Risk Stratification of Competing-Risk Model

**FIGURE 4:**
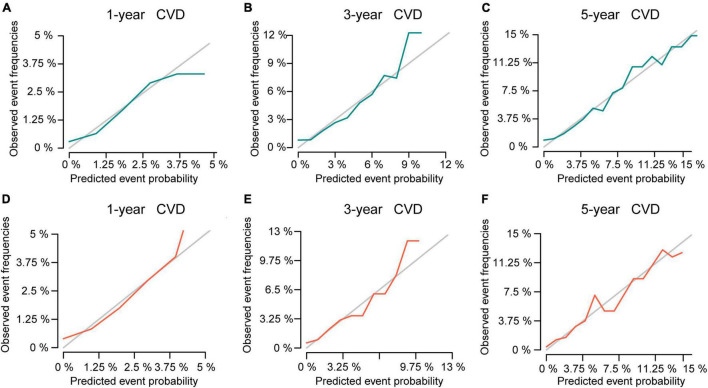
The calibration curve for predicting the 1-, 3-, and 5-year probabilities of CVD in the training **(A–C)** and the validation cohort **(D–F)**, respectively.

**FIGURE 5:**
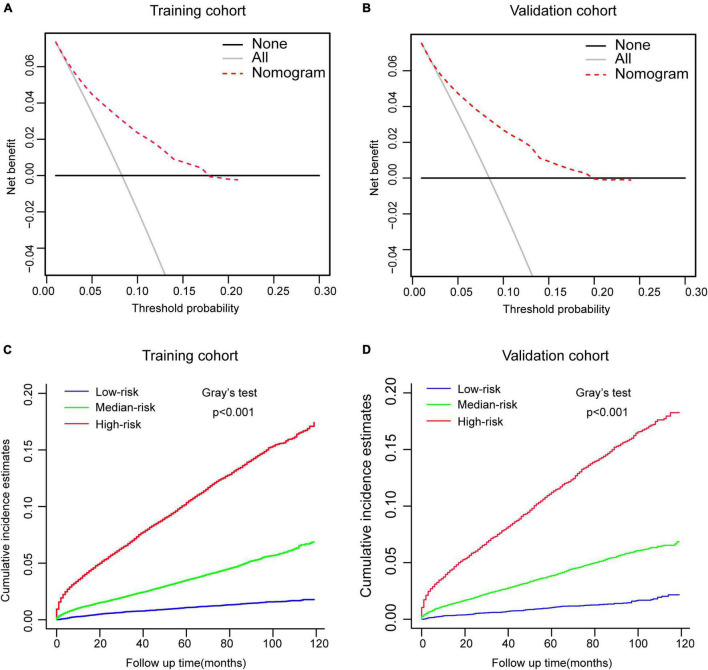
Performance and risk stratification of the competing-risk model. **(A,B)** Decision curves of the nomogram predicting CVD in the training and validation cohort, respectively. **(C,D)** Cumulative incidence function (CIF) curves with the *p*-value of Gray’s test for the training and validation cohort, respectively.

Then, this nomogram was validated using bootstrap and ten-fold cross-validation methods. The results showed that nomogram had a great discrimination ability in predicting overall survival (OS), with a C-index of 0.719 (95% CI, 0.738–0.742), and 0.719 (95% CI, 0.622–0.668) in the training and validation cohort, respectively. The calibration curves were shown in Figure 4, with the dots close to a 45° diagonal line, reflecting great consistency between the prediction by the nomogram and the actual observation of the probability of CVD at 1-, 3-, and 5-year. Furthermore, DCA was introduced to assess the clinical utility of the nomograms. As shown in Figures 5A,B, the clinical use of the nomogram showed high positive net benefits at a wider range of threshold likelihood, which depicted that the nomogram had a high clinical utility in predicting CVD.

According to the tertile values of the nomogram-based scores derived from the training cohort, the patients were categorized into high-risk, medium-risk, and low-risk groups in both cohorts. The high-risk group had the highest probabilities of CVD, followed by the medium-risk group and the low-risk group in both cohorts (Figures 5C,D). Hence, there is an important value of competing-risk model for clinical risk stratification and prognosis decision in patients with CRC.

## Discussion

In the past few decades, considerable advances in management of cancer have greatly prolonged the survival of patients suffering from CRC. On the other hand, we can also expect that non-cancer mortalities will become more prevalent, dominated by cardiovascular disease. Based on the SEER database, our current study provided important insights into the risk of CVD among patients with CRC diagnosed between 2007 and 2015. In total, 79,455 patients died throughout the follow-up, of whom 56,185 (70.71%) succumbed to CRC and 23,270 (29.29%) died due to non-CRC, among which CVD accounted for 9,702 (41.69%), being the major cause of non-cancer deaths. The 1-, 3-, and 5-year CIF of CVD were 12.20, 24.25, and 30.51%, respectively, while the 1-, 3-, and 5-year CIF of no-CVD were 1.93, 4.13, and 4.77%, respectively, indicating that CVD has become a main reason of death among CRC survivors during the follow-up period. Through competing risk analyses, we further identified that age, race, marital status, tumor size, SEER stage, surgery, and chemotherapy were independent risk factors for CVD. These results should not be ignored when evaluating the individual risks of CVD and work as an indication for more precise treatment and risk factors management, such as monitoring of blood sugar and hypertension and health education.

Currently, chemotherapy and widely used drugs for CRC, which involves several agents, such as oxaliplatin, fluorouracil, leucovorin, and so on (25, 26), are effective. Chemotherapy usually induces cardiotoxicity and increases CVD risk (27). In addition, drugs for CRC usually lead to higher CVD risk than the general population, particularly in the first few years of treatment (28). However, our analysis indicated that the risks of CVD were significantly lower among patients with CRC who were treated with chemotherapy than those were not. This result seemed to be contrary to the observed cardiotoxic effect of chemotherapy. However, consistent with the previous studies in other tumors (29, 30), this contradiction arises from the limited life expectancy of patients who received chemotherapy and succumbed to CVD events. Since the detailed information for chemotherapy regimen were missing in the SEER database, further investigation is required to clarify the effect of chemotherapy on the risk of CVD among patients with CRC. In addition, we also demonstrated that patients without cancer-direct surgery had an increased CVD compared to patients who received surgery, which was in line with previous findings (13, 31).

In recent years, the role of socioeconomic factors in influencing humans, including cultural and social values, insurance status, education level or employments status, and so on, are increasingly becoming the focus of medical attention (32, 33). In this study, we investigated the effects of insurance and marital status on the risks of CVD. Results showed that the insured patients had lower risk of CVD compared with those who were uninsured. For now, battling CRC has been regarded as a time-consuming, multidisciplinary, and expensive process. Uninsured patients usually suffer the brunt of shortage of medical services and supplies. Furthermore, we demonstrated that, among CRC survivors, marital status was a protection factor against CVD. Marital status is a potential marker of mental status, lifestyle, and social and family support, which have greatly affected the outcomes of patients with cardiovascular disease (34). Patients who are married display less distress and anxiety than their unmarried counterparts after a diagnosis of cancer (35–37), and this could contribute to increased family support, medication compliance, and survival advantages to a large extent. In addition, from the perspective of biological factors, a married status benefited to promote cardiovascular, endocrine, immune status, and nutrition behavior (34, 38). Collectively, we strongly recommend the integration of non-biological factors when assessing the individual risks of CVD among CRC survivors.

To facilitate patient counseling and clinical decision making, a prospective risk of potential cardiotoxicity for individual is imperative. From the clinical perspective, we constructed a competing-risk model with variables to investigate the probabilities of CVD at 1-, 3-, and 5-year. To the best of our knowledge, this is the first study that established and validated a competing-risk model based on the Fine-Gray proportional subdistribution hazard analysis to estimate the individual probabilities of CVD-specific mortality for patients with CRC. All the variables included in this nomogram were easily available in clinical practice. With its aid, clinicians can more expediently devise clinical managements and, more importantly, remain vigilant for this complication when treating patients with CRC with immunotherapy. Our nomograms showed excellent accuracy and discriminative performance, as validated by C-index and calibration curves. Furthermore, we should be aware that high discrimination calibration does not necessarily imply an excellent clinical utility. Hence, DCA was employed to determine the clinical utility of this nomogram by calculating the net benefits at each risk threshold probability (23, 24). Results showed that using the nomogram to predict the probabilities of CVD provided more benefits. Collectively, these data demonstrated that this model had strong practicability and high reliability in the processes of clinical practice.

The major advantages of this study were that it had a large enough sample size and that it used competing risk analysis to investigate the risk of CVD among patients with CRC. Generally speaking, the SEER database, accounting for about a third of the United States population, provides large enough sample data to explore risk factors and further develop a nomogram based on competing risk analysis. More to the point, results from analyses that use population-level databases tend to be more generalizable and representative than those from single-center reports (24). Actually, sufficient incorporated samples are needed to guarantee the accuracy of nomograms, as demonstrated our recent publications (5, 18, 24). Notably, no competing-risk model has been established to evaluate the risk of CVD among patients with CRC so far. We established the first competing-risk model for these patients and made possible the individualized prediction of prognosis. Furthermore, our nomogram showed excellent discrimination power and clinical usefulness in clinical practice. In addition, the parameters included in the nomogram could be easily obtained in clinical practice.

Undoubtedly, this study was subject to several limitations. First, it had a retrospective design, making potential hidden biases. In addition, there is no way to know some the relevant information, such as gene mutations (HER-2 and RAS/RAF), making it impossible to adjust for these characteristics between the two groups. Second, the SEER database did not provide an explanation about comorbidity since it was a significant factor when physicians deciding treatment strategies. This lacking would, to certain degree, weaken the objectivity of our conclusions. In addition, it remains a main limitation that we established a model without comorbidity status. Third, data on chemotherapy regimen were not available in the SEER database, and some of which is closely associated with cardiotoxicity. Finally, although the competing-risk model had excellent performance in predicting the probabilities of CVD, it was validated by an internal patient cohort. Thus, additional external data is needed to verify the performance of the model further.

## Conclusion

The present study was the first to use a competing-risk model to investigate the cumulative incidence and risk factors of CVD among patients with CRC. More importantly, we have successfully developed a nomogram for predicting the probabilities of CVD for patients with CRC. The internal and external validation demonstrated the excellent discrimination, calibration, and clinical usefulness of this model. With the help of this well-established nomogram, clinicians would make more individualized treatments, tighter control of modifiable risk factors, and follow-up schedules.

## Data Availability Statement

The datasets presented in this study can be found in online repositories. The names of the repository/repositories and accession number(s) can be found in the article/supplementary material.

## Ethics Statement

Approval was waived by the Local Ethics Committee, as Surveillance, Epidemiology, and End Results (SEER) data is publicly available and de-identified.

## Author Contributions

TL and SZ were responsible for the conception and design of the study, assisted with the statistical analysis, and wrote and revised the manuscript. YW, PZ, and LA contributed to the data analysis and revision on the English language and grammar, and corrected the parts of the discussion. TL made the primary contribution in the later stage in the writing and modification of the manuscript, and review of the finalized article. All authors contributed to the article and approved the submitted version.

## Conflict of Interest

The authors declare that the research was conducted in the absence of any commercial or financial relationships that could be construed as a potential conflict of interest.

## Publisher’s Note

All claims expressed in this article are solely those of the authors and do not necessarily represent those of their affiliated organizations, or those of the publisher, the editors and the reviewers. Any product that may be evaluated in this article, or claim that may be made by its manufacturer, is not guaranteed or endorsed by the publisher.
